# Control of a Humanoid NAO Robot by an Adaptive Bioinspired Cerebellar Module in 3D Motion Tasks

**DOI:** 10.1155/2019/4862157

**Published:** 2019-01-27

**Authors:** Alberto Antonietti, Dario Martina, Claudia Casellato, Egidio D'Angelo, Alessandra Pedrocchi

**Affiliations:** ^1^Department of Electronics, Information and Bioengineering, Politecnico di Milano, Milano, Italy; ^2^Department of Brain and Behavioral Sciences, University of Pavia, Brain Connectivity Center Istituto Neurologico IRCCS Fondazione C. Mondino, Pavia, Italy

## Abstract

A bioinspired adaptive model, developed by means of a spiking neural network made of thousands of artificial neurons, has been leveraged to control a humanoid NAO robot in real time. The learning properties of the system have been challenged in a classic cerebellum-driven paradigm, a perturbed upper limb reaching protocol. The neurophysiological principles used to develop the model succeeded in driving an adaptive motor control protocol with baseline, acquisition, and extinction phases. The spiking neural network model showed learning behaviours similar to the ones experimentally measured with human subjects in the same task in the acquisition phase, while resorted to other strategies in the extinction phase. The model processed in real-time external inputs, encoded as spikes, and the generated spiking activity of its output neurons was decoded, in order to provide the proper correction on the motor actuators. Three bidirectional long-term plasticity rules have been embedded for different connections and with different time scales. The plasticities shaped the firing activity of the output layer neurons of the network. In the perturbed upper limb reaching protocol, the neurorobot successfully learned how to compensate for the external perturbation generating an appropriate correction. Therefore, the spiking cerebellar model was able to reproduce in the robotic platform how biological systems deal with external sources of error, in both ideal and real (noisy) environments.

## 1. Introduction

This work belongs to neurorobotics, a discipline that has the objective to replicate typical animal behaviours in robotic platforms. Its aim is to develop systems that, through specific algorithms and computational models inspired by biology and physiology, are capable of mimicking the sensory and motor control mechanisms of animals and humans. This ambitious objective is pursued in order to develop a better understanding of the biological mechanisms that rule our behaviours. The obtained technology and systems will also provide valuable feed-back and feed-forward control functions that could introduce sensory-motor coordination in robots. In this work, we focused on a bioinspired cerebellar simulator integrated into the controller of a humanoid robot. We tested its learning properties in the typical sensorimotor task of perturbed upper limb reaching [1].

Motor control is one of the main tasks of the central nervous system (CNS), and many hypotheses on its operating principles and mechanisms have been proposed. Considering the intimate relation between the motor control system and the sensory system in the motor execution, it is possible to refer to their combined behaviour as a sensorimotor loop. This loop combines both feed-forward and feed-back strategies where the sensory and cognitive processes are the inputs that generate the next motor output. Computationally, the CNS is represented by the system that processes the inputs and generates the outputs. The inputs consist of all the sensory information from external and proprioceptive receptors as well as the cognitive internal signals. The output is the motor command directed to the muscles which will produce an effect on the environment. The sensorimotor loop comprehends many different substructures and, among the others, the cerebellum. It has a major role in the fine motor control and in the learning of motor tasks and association patterns. The cerebellum processes the data from many sensory channels (i.e., vestibular and proprioceptive) and combines them with the previous motor commands to produce the updated motor commands for the next execution. The cerebellum is also supposed to be involved in a large amount of cognitive learning processes [2]. The paradigms assessed in literature, stressing the cerebellar role, are the eye blinking classical conditioning for the associative tasks [3], the vestibule-ocular reflex (VOR) [4], and the perturbed upper limb reaching for the motor control [5].

More than half of all the brain neurons are located in the cerebellum, which accounts for just the 10% of the brain mass. The cerebellar cells are thus densely packed in the grey matter of the highly convoluted cerebellar cortex and in the four deep cerebellar nuclei, on both brain hemispheres. The cerebellar cortex is composed of three layers, which include at least five types of cells. The cerebellar structure and its functional behaviours have been deeply analysed. During this extensive study, many cerebellar computational models have been proposed and developed [6–9]. Among these, the phenomenological models obtained from computational motor control studies are the best candidates to solve the sensorimotor integration issue, since they use an abstract function-based approach. This kind of models is capable to deal with motor planning, internal models, state estimation, motor learning, and modularity [10]. A realistic approach based on neurobiology requires the use of interconnected adaptive spiking neural networks (SNN). These networks have the potential to reproduce good adaptive control systems for robots, considering how biological organisms perform excellent control using ensembles of interconnected neurons [11].

In previous works, we have tested a range of simplified, but realistic, cerebellar models into sensorimotor tasks [12–14]. The objective of this work was to implement a computational model in a robotic platform in order to validate its functionality and behaviour in real-time control problems. In particular, a robot controller has been integrated with the cerebellar-inspired network, which provides the system with the capability of motor learning. The component of the motor control system can be represented by their phenomenological models, such as the feed-back and feed-forward mechanisms of the internal models. The first is based on the ongoing error coming from the sensor updates, while the latter is based on the direct motor commands. To allow such integration between the robotic platform controller and the bioinspired spiking network, we needed to introduce the right interfaces within the modules. Three interfaces have been introduced; two that encode the desired inputs trajectories and the errors obtained into a spiking activity, and the other one that decodes the frequency of the output neurons into an angular value that can be then applied by the robot controller. The spiking neural network has been simulated by the EDLUT platform, a SNN simulator which allows a real-time execution. By means of look-up tables, EDLUT bypasses the need to solve the differential equations governing the state of every network unit, thus reducing the computational load.

## 2. Materials and Methods

First, we defined a suitable version of the perturbed upper limb reaching protocol. This paradigm is used to enhance the cerebellar effect in the sensorimotor loop. The objective is to have the subject, the robot in this case, to follow a particular trajectory, At a certain point, an unexpected external force is applied which will then produce a perturbation in the performed trajectory. The same perturbation is then applied in a sequence of trials, so that the subject learns to predict the perturbation and thus to limit the error (acquisition phase). After that, the perturbation is suddenly removed, the subject generates an error in the opposite direction, which is canceled out in the following trials (extinction phase).

The network was tuned using a robot simulator to perform a physiological behaviour; therefore, we set the starting weight values between the different kinds of cells to provide activity frequencies matching the ones present in the literature. Then, we performed a brute force exploration to find the best combination of the model plasticity parameters.

Once the network was optimized, we proceeded to test its capability of generalization with other trajectories, different than the one used to train the network. To further investigate the network behaviour, we evaluated its performances with the real robot using the parameters derived from the tuning with the simulator. We evaluated how the different plasticities and the gain tuning affected the performances on a noisy environment and also how the network in the real robot can deal with the different perturbations (i.e., different trajectories).

A final test was performed using a network ten times larger, with the same trajectory and parameters, to verify how a more detailed SNN could alter the performances for the proposed motor task.

Summarizing, we have focused this work on three main objectives: (i) the optimization of the upper limb reaching protocol, adapting it to the NAO robot to simultaneously control 3 degrees of freedom (DoFs); (ii) the parameter tuning of the SNN, to replicate the physiological behaviour of its constituent neurons and of the resulting robot behaviour; and (iii) the exploitation of the optimized network on different trajectories (transfer learning).

### 2.1. Simulated and Real Robots

**Figure 1:**
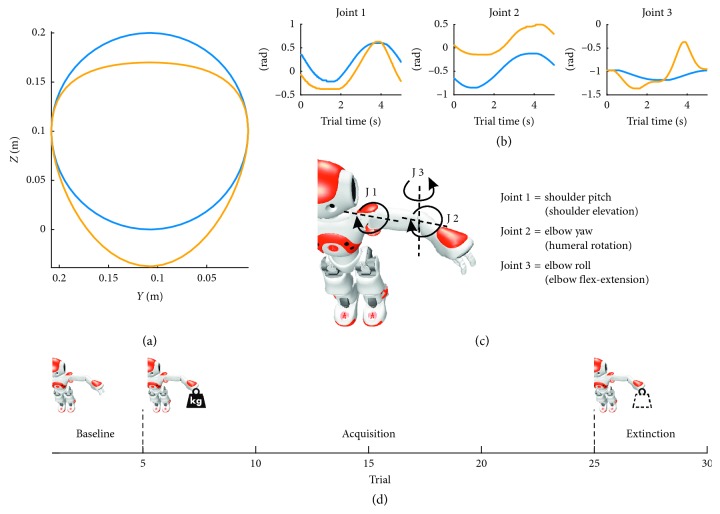
Trajectories and experimental protocol. (a) Planar representation (*Y*-*Z* axis, in the robot reference frame) of the ideal (blue) and perturbed (yellow) Cartesian trajectories. The corresponding trajectories in the joint space are depicted in panel (b). (c) The controlled joints of the robot correspond to three rotations: shoulder elevation (Joint 1), humeral rotation (Joint 2), and elbow flex extension (Joint 3). (d) The experimental protocol consists of 5 baseline trials, 20 trials of acquisition, where a load is applied to the robot arm, and 5 trials of extinction, where the additional load is removed.

NAO is an integrated, programmable, medium-sized humanoid robot developed by Aldebaran Robotics. NAO (version V3.3) is a 58 cm, 5 kg robot, communicating with remote computers via an IEEE 802.11 g wireless or a wired Ethernet link. The NAO has 21 DoFs and features a variety of sensors. Since we were interested in arm movements, we controlled three joints of the robot left shoulder and elbow (Figure 1(c)): shoulder pitch (Joint 1), elbow yaw (Joint 2), and elbow roll (Joint 3).

To interface the robot with the neural network, able to run in real time with a frequency of 1 kHz, a stable frequency update was a strict requirement. For this reason, the robot was commanded by means of the device communication manager (DCM). The DCM is the software module that is in charge of the communication with all electronic devices in the robot (boards, sensors, actuators, etc.). It is the link between high-level functions and low-level controllers. The DCM has a separate real-time thread that runs every 10 ms, thus guaranteeing a stable refresh rate of 100 Hz.

NAO robot cannot be controlled directly in torque/current to the motors, but only in position. This forbids to physically perturb the arm reaching motion task with an external force. In case of a lower force, the robot arm would just reach the desired angle, while in case of a higher force the motor would stall without moving at all. For this reason, two different trajectories were used: one ideal trajectory used as the desired path and another *perturbed* trajectory, to be corrected by the network using the angular errors of the joints as learning signals.

Since the network optimization process will need hundreds of tests, we used a robot simulator called Webots. This simulator allows launching a simulated NAO moving in a virtual world, offering a safe place to test behaviours before deploying them on a real robot. Considering the level of control offered by the DCM and the unpredictable behaviour of the SNN, especially in tuning phase, a simulator was ideal to test how the motor commands would be affected and to prevent dangerous commands to be sent to the real robot. While being a very accurate simulator for the NAO, as with any other simulator, some nonidealities are not considered (e.g., nonlinear friction, sensor errors, and motor overheating).

### 2.2. Cerebellar Model

**Figure 2:**
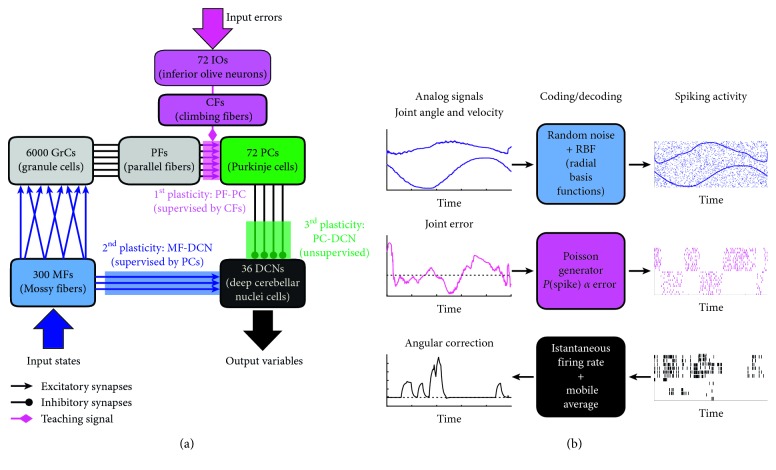
Cerebellar SNN and coding/decoding strategies. (a) The computational model applied for creating the cerebellar SNN embedded into the controller of NAO robot. Each block represents a neural population, with the relative inputs and outputs. The excitatory, inhibitory, and teaching connections are depicted. The shaded areas represent the three plasticity sites: magenta the PF-PC synapses, blue the MF-DCN synapses, and green the PC-DCN synapses, adapted from [15]. (b) Coding (for MFs and IOs) and decoding (for DCNs) strategies implemented to integrate the analog robotic world with the spiking activity of the SNN. The 3 joint angles and angular velocities are fed as input to the MFs by means of an RBF approach, overlapped to a random activity. Each joint error is transformed into IO spikes by means of Poisson generators, which produce spikes with a probability that is proportional to the error magnitude. Each IO generates a spike pattern that is therefore independent of their history and of the other IOs. The DCN spikes are transformed into an angular correction sent to the robot joints by means of an instantaneous firing rate computation, subsequently averaged with a mobile-window filter.

In this work, a cerebellar-inspired SNN, based on previous versions presented and tested in [12, 13], was used to prove its adaptation capabilities in a complex motor task. The cerebellar neural network used has the following architecture (Figure 2(a)), built taking inspiration from physiological studies of the cerebellum, in a tight collaboration with neuroscientists. The SNN was composed of 6480 Leaky Integrate and Fire neurons replicating the cerebellar microcircuit: 300 mossy fibers (MFs), the first input of the cerebellar network, organized in 6 groups of 50 cells each: 3 groups, one for each controlled joints, encoding information on the desired positions and 3 groups encoding information on the desired velocities; 6000 granular cells (GrCs), generating a sparse representation of the state input; 72 inferior olive neurons (IOs), the second cerebellar input, with their respective climbing fibers (CFs); IOs are divided into 6 groups of 12 cells each, 3 groups, one for each joint, encoding the positive errors and other 3 groups encoding the negative ones; 72 Purkinje cells (PCs), the integrators of the sparse state-information coming from the GrCs through the parallel fibers (PFs) with the error-information coming from the IOs through the CFs; 36 deep cerebellar nuclei neurons (DCNs), which are the only output of the cerebellar microcomplex, thus producing the cerebellar output variable, divided in 6 groups, two for each joint, where one group controls the compensation of positive errors (i.e., agonist muscles) and the other compensates for negative errors (i.e., antagonist muscles).

Three interfaces (Figure 2(b)) were implemented to transform analog signals into spiking activity to be fed to the network (input) and vice versa (output).

The first interface computes the input current for MFs with a radial basis function (RBF) method. This current *I*(*t*) is used to increment the membrane potential *V*_*m*_(*t*) of the MF. RBF centers are distributed equally along the sensory dimensions, with their widths tuned to ensure a small overlap in the response of consecutive MF. One-dimensional values are converted into multidimensional current vectors, one for each RBF. Every MF has its own receptive field to encode the analog information, normalized between −1 (minimum value) and 1 (maximum value).

The second interface converts joint errors into IO spikes. These neurons have a low firing rate (less than 10 Hz) that could prevent the representation of high-frequency error signal related to the task being learned. This issue can be fixed exploiting the irregular firing of the IO by statistically sampling the entire error signal over multiple trials. It has been observed that the temporal distribution of the spikes of IOs shares similar characteristics as the Poisson model. IOs fire randomly in behaving animals at rest and during the ocular following response and arm-motion tasks in monkeys. This stochastic characteristic firing enhanced the input-output mutual information despite the ultralow firing rates of CFs [16]. The firing rate is reproduced with a Poisson model of spike generation and, at every time step, IO spikes are randomly generated with a probability that is proportional to the error magnitude. This approach has been employed to generate independent spike patterns on multiple IO neurons. For each IO, the firing probability is therefore independent of the previous spiking activity and from the activity of the other IOs [17, 18]. Substantial evidence supports a role for CFs in error signalling and motor learning, and the proportionality between the error signal and the spiking activity of CFs has been verified [19, 20].

The third interface decodes DCN spike patterns into analog angular values. First, the instantaneous firing rate of every DCN is calculated, and then positive and negative DCN firing rates are averaged with a mobile time window of 200 samples (i.e., 200 ms). The cerebellar output is obtained by calculating the net difference between the two averaged mean DCN firing rates (positive and negative) [12].

Thanks to these interfaces, the SNN could be integrated into the robotic platform controller, with the function of a feed-forward predictive controller.

The SNN neurons are connected in three possible ways:

- Excitatory connections: synapses in which an action potential in a presynaptic neuron increases the probability of an action potential occurring in a postsynaptic cell. There are excitatory connections between MFs and GrCs, between MFs and DCNs, and between GrCs and PCs.
- Inhibitory connections: synapses in which the impulse in a presynaptic cell results in a reduced likelihood for a postsynaptic cell to produce an action potential. There are inhibitory connections between PCs and DCNs.
- Teaching connections: connections that encode teaching spike trains (related to the error) for the supervised learning in plasticity sites of the cerebellum.

According to neurobiological studies, three plasticity sites have been identified in the human cerebellum: at the level of PF-PC excitatory connections; at the level of MF-DCN excitatory connections; and at the level of PC-DCN inhibitory connections [21–24].

The SNN model was equipped with three plasticity sites, at cortical (PF-PC) and nuclear (MF-DCN and PC-DCN) levels. The synaptic connections in each site followed three different learning rules, which strengthen or weaken these connections by long-term modifications: long-term potentiation (LTP) and long-term depression (LTD). LTP and LTD mechanisms were modeled as modifications on the synaptic conductances as described in detail in [13, 15]. In general, the three mechanisms were based on different kinds of Spike-Timing-Dependent Plasticity (STDP), but each one was tailored to the specific experimentally measured mechanism. The first plasticity (PF-PC) modulates the activity of PCs, increasing or decreasing the synaptic strength of the connections under the supervision of the IO activity. The second plasticity (MF-DCN) is also a supervised learning rule; in this case, the PC activity is the modulator signal that influences the synaptic weights. The third plasticity (PC-DCN) is an unsupervised standard STDP, where the weight modifications are driven uniquely by the timing of the presynaptic (PC) and postsynaptic (DCN) neurons.

For the initialization of the network synaptic weights, we referred to physiology values. MF activity has been set to a frequency comprised around 50 Hz, by adjusting the background random activity and the overlap and bell width of the RBFs. MF-GrC weights have been set to achieve a GrC frequency of 3–6 Hz and the GrC-PC weights to produce a PC frequency around 40–60 Hz. MF-DCN weights were set in order to have a DCN frequency around 25 Hz in absence of PC inhibition (PC-DCN weight = 0). The last step consisted in adjusting PC-DCN weights, nullifying the DCN activity in presence of a stable PC activity around 45 Hz.

To perform the simulations in real time, we leveraged the EDLUT simulator [25], an open source simulator of SNN that provided a reduction of the computational loads, speeding up the network simulation by means of look-up tables. In fact, with a standard simulator (e.g., NEURON [26], NEST [27, 28], or Brian [29]), the program has to solve one or more differential equations for each neuron and cannot guarantee the real-time performances that are required in interfacing a real robotic platform.

### 2.3. Experimental Protocol

We challenged the SNN in a 3D motion adaptation protocol, similar to adaptation protocols based on force-fields performed on human subjects [30–32]. The ideal trajectory that the robot wants to perform is a planar circle of 0.1  *m* radius, executed in the *Y*-*Z* plane and with center *Y*=0.1 *m* and *Z*=0.1 *m* (Figure 1(a), blue line). When an unexpected load is virtually added to the robot hand, the trajectory deviates from the desired one, being deformed toward the ground (Figure 1(a), yellow line). As a result, the three controlled joint angles deviated from the ideal paths (Figure 1(b)), thus generating positive and negative errors for each DoF.

The experimental protocol consisted in 30 trials divided into three phases (Figure 1(d)): the first one was the baseline phase, in which the command for the robot was the ideal trajectory and lasted for 5 trials. The second phase was the acquisition phase that lasted for 20 trials and in which the input for the robot was the perturbed trajectory. The last one was an extinction phase of 5 trials, in which the input was again the ideal trajectory. In order to mimic the adaptation capabilities of the cerebellum, the goal of the SNN was to minimize the joint errors, thus reducing the subsequent Cartesian errors in the 3D space.

### 2.4. Parameter Tuning

As mentioned above, there are three different plasticity sites that can modify the behaviour of the SNN, each one is characterized by two learning parameters: LTP and LTD. In order to assess the best values for these six parameters, a brute force exploration has been performed. The first plasticity (cortical plasticity, PF-PC) is the main cause of the learning effect, as it regulates the activity of the PC which depresses the DCN output. The other two plasticities (nuclear plasticities, MF-DCN, and PC-DCN) have a secondary effect, affecting the error reduction performance on a longer time scale and with a lower magnitude. The parameter tuning tests have been performed with Webot simulator, to prevent damages and avoid unpredictable movements of the robot arm due to unexpected behaviour of the network.

To evaluate the network performance, we calculated a global cost function, which we wanted to minimize. The cost function is the sum of two quality metrics. Both metrics take into account the root mean square error (RMSE) of all the three joints. For each trial, RMSE for every joint was computed over the 5000  *ms* of trial time, and then the three RMSEs were averaged. The average RMSE for the *i* ^th^ trial is computed as

(1) $$\begin{array}{c} {\text{RMSE}}_{\text{Avg}}\left(i\right)=\frac{{\text{RMSE}}_{\text{Joint}1}\left(i\right)+{\text{RMSE}}_{\text{Joint}2}\left(i\right)+{\text{RMSE}}_{\text{Joint}3}\left(i\right)}{3}. \end{array}$$

The first quality metric computed a weighted average of RMSE_Avg_ in the acquisition and extinction phases. While it is normal to have higher errors in the first acquisition trials, a good cerebellar controller should gradually correct the ongoing joint errors. This metric rewards SNN showing a good correction in the late stages of the acquisition and also low extinction errors:

(2) $$\begin{array}{c} {\text{RMSE}}_{\text{Weighted}}=\frac{\sum_{i=6}^{30}{\text{RMSE}}_{\text{Avg}}\left(i\right)\cdot \text{weight}\left(i\right)}{25}, \end{array}$$

where

(3) $$\begin{array}{c} \text{weight}\left(i\right)=\left\{\begin{array}{cc} \frac{i-4}{3}, & \text{if }6\leq i\leq 25\;\;\left(\text{acquisition phase}\right),\\ 4, & \text{if }i>25\left(\text{extinction phase}\right). \end{array}\right. \end{array}$$

The second quality metric measures the stability of the correction, computing the standard deviation (SD) of the trials 21–25 (i.e., the last 5 trials of acquisition). High values of LTP and LTD parameters could lead to fast changes in the RMSE in the acquisition phase, but also to its instability. This leads to a high standard deviation of the RMSE, especially in the last five trials, where the minimum error should have been reached already:

(4) $$\begin{array}{c} \text{STD}=\frac{\sqrt{\sum_{i=21}^{25}{\left({\text{RMSE}}_{\text{Avg}}\left(i\right)-\bar{{\text{RMSE}}_{\text{Avg}}}\right)}^{2}}}{5}. \end{array}$$

Finally, a global cost function, whose minimum should identify the most performing model in the explored area, is calculated normalizing RMSE_Weighted_ and STD over their maximum and minimum values, thus obtaining values between [0-1], and then summing them to obtain a global cost value in the range [0–2].

For the first plasticity, we evaluated an 11 × 11 matrix, with LTP1 values ranging from 0 to 0.01 with steps of 0.001 and LTD1 values ranging from 0 to 0.05 with steps of 0.005. The exploration was performed iteratively, choosing an LTP value and pairing it with all the LTD values and then repeating the process for all the other LTP values. For each LTP and LTD combination, a complete simulation of the protocol was performed, and the final global cost value was computed. After the first exploration, a second one has been performed in the area of the global minimum of the cost function, with finer steps, testing other 10 × 10 values. The best LTP-LTD configuration has then been chosen for the tuning of the other nuclear plasticities.

For the nuclear plasticities, the LTP1 and LTD1 resulting from the previous exploration have been kept fixed, and the exploration has been performed on LTP and LTD parameters (i.e., LTP2, LTD2, LTP3, and LTD3). The evaluation was similar to the first plasticity, with the exception of the parameter ranges. LTP and LTD ranged from 10^−10^ to 10^−1^ with a ×10 steps. As before, a second exploration in the best area identified was performed. The second search covered a 10 × 10 parameter area centered on the best parameter identified in the first exploration, testing half of the below and above values (i.e., $\left[\begin{array}{cccccccccc} 0.5 & 0.6 & 0.7 & 0.8 & 0.9 & 1 & 2 & 3 & 4 & 5 \end{array}\right]\times 10^{j}$, where *j* is the best generation exponential).

Once the plasticities values have been set, another parameter to consider is the gain needed to convert the analog output of the network in an angular value (in radians). Since each joint has different ranges and different errors amplitude, a proper gain for each joint has to be used. To find the optimal gain values, a brute force exploration has been performed with a gross exploration (i.e., testing gain values ranging from 0.005 to 0.05 with steps of 0.005) and a subsequent finer exploration (i.e., testing 10 gain values centered on the best result of the gross exploration, with steps of 0.001). The gain factor is particularly relevant due to the normalization of the angular values and the error that is given as input to the network. As the network manages values comprised between 0 and 1 for all joints, its output does not consider the differences in the actual angular errors. Therefore, a joint with small angular error will require a lower gain, while more perturbed joints will require a greater gain.

### 2.5. Transfer Learning

Having identified the set of the network parameters (i.e., LTP1 − 3, LTD1 − 3, and gain  1 − 3) which produced the best performances, we have verified if (i) the SNN was able to compensate for an external perturbation a 3D movement performed by a physical NAO robot and if (ii) the SNN was able to compensate different perturbations on both simulated and physical robotic platform.

Therefore, we executed 10 tests with both Webot simulator and NAO robot, in order to verify the robustness of our controller in a noisy system performing the same protocol used for the SNN optimization.

To verify the transfer learning capabilities of the cerebellar model, we have executed 10 tests with both Webot simulator and NAO robot in variations of the protocol, with 3 other couples of ideal and perturbed trajectories: two deformations of the ideal circle trajectory (an oval and a squared deformation) and with ideal and deformed *∞*-shaped trajectories.

### 2.6. Network Enhancement

One of the limits of the network used is the low number of output cells which limits the resolution of the correction, leading to jerky angular trajectories. Therefore, we tested an expanded version of the SNN, to observe how the network size can change the overall performances of the cerebellar-inspired controller. Each neural population was increased ten-fold, maintaining the same connectivity rules explained above. All parameters have been kept the same as the ones used with the normal-sized network, and the gain has been reduced by ten, in order to match the increased number of output cells. A side effect of the increased size of the network is the loss of the real-time property due to a larger number of spikes to be processed. Therefore, the larger network was tested for a single test instead of the usual 10 tests. Also in this case, we tested also the three additional trajectories to verify the generalizability of the learning properties of the SNN.

## 3. Results and Discussion

### 3.1. Parameter Tuning

After every plasticity gross exploration, the parameter space area resulting in the best performance, according to the developed cost function, was further explored. The RMSE over all trials was computed for 10 tests, using the parameters found during the optimization, in order to verify the behaviour of the SNN over the different phases.

**Figure 3:**
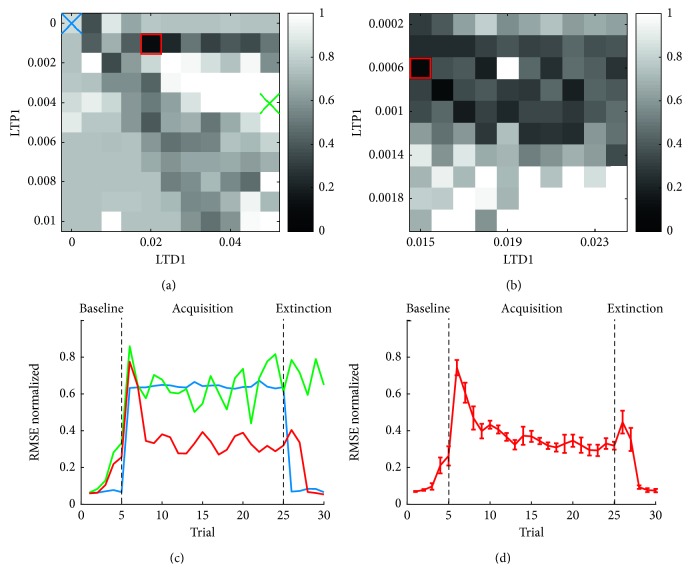
Cortical plasticity optimization. (a) Cost function resulting from the gross exploration of LTP1 and LTD1 parameters. Darkest values represent low values of the cost function, therefore the best combinations of the two plasticity parameters. The parameter space further explored in the finer search (b) is identified by the red square. Blue and green crosses identify two examples parameters giving bad performances (c). (b) Cost function resulting from the finer exploration of LTP1 and LTD1 parameters. The red square identifies the global minimum, therefore the chosen combination of LTP1 and LTD1. (c) Three examples of RMSE performance across the 30 trials of the protocol. The red line represents a good performance, with a reduction of the RMSE during the acquisition phase and a good extinction in the last 5 trials. The blue line represents the combination of LTP1=0.0 and LTD1=0.0; therefore, no correction happened in the acquisition phase, leading to a high cost function value. The green line represents a combination of too high LTP1 and LTD1, leading to an unstable and ineffective correction along the trials. (d) Mean and SD of the RMSE in 10 tests performed with the Webot simulator with the best combination of LTP1 and LTD1 identified in the finer exploration.

In Figure 3(a), it is possible to see the effect of LTP1 and LTD1 on the quality of the correction: there are three main areas that is possible to identify. The first one is the lower-left corner, where LTD is too low and LTP is consistently higher (blue cross). Since the LTD is the major player in the attenuation of PC activity and the consequent increase of DCN activity, the network does not perform well in the acquisition phase (i.e., there is no learning) while it performs well in the extinction phase as there is no overcompensation when the additional load is removed (Figure 3(c), blue line). The second area is the middle right corner, where LTD is higher than LTP (green cross). Here the activity of the DCN reaches high levels, and since the correction is strong, this is the worst area for the extinction phase. Note that also in acquisition, this area is not useful, as a too fast and aggressive correction leads to instability in the final trial of this phase (Figure 3(c), green line). Finally, there is the area around the principal diagonal, where we can find lower values of the cost function. The top central area containing the global minimum (red square) is the area chosen for the finer exploration (Figure 3(b)).

The finer exploration produced more uniform results, but it was still possible to exclude the lower area with too high values of LTD, where the correction was insufficient and unstable. The global minimum of this exploration corresponded to the parameters LTP1=0.0006 and LTD1=0.015. As already proved in previous works [21–23, 33], the cortical LTD has greater values with respect to the LTD. This combination of parameters was tested ten times to assess the reproducibility of the results (Figure 3(d)), and it was then used during the tuning of the nuclear plasticities.

**Figure 4:**
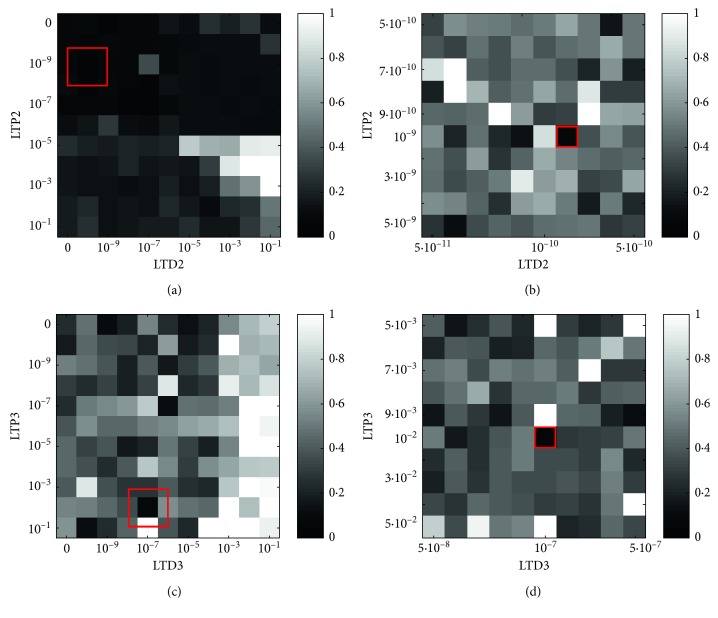
Nuclear plasticities optimization. (a, b) Cost functions resulting from the gross and finer explorations of LTP2 and LTD2 parameters. Darkest values represent low values of the cost function, therefore the best combinations of the two plasticity parameters. The parameter space further explored in the finer search (b) is identified by the red square (a). (c, d) As (a, b), but for the gross and finer exploration of LTP3 and LTD3.

The results obtained after the second plasticity (MF-DCN) gross exploration are shown in Figure 4(a)). It is evident that the highest errors are present in the bottom right area, where the high LTP produce an overcompensation effect. Note that, for this plasticity, LTP2 is the main responsible for the increased activity of the DCN while LTD2 concurs to their attenuation. In the right area, too high LTD2 leads to the absence of DCN activity and therefore of the correction. In the left area, the LTD2 allows an activity from the DCN, and thus the better results. We investigated the top left area, containing the global minimum. The finer exploration (Figure 4(b)) was almost uniform; therefore, we identified the minimum global cost function for the combination of parameters LTP2=10^−9^ and LTD2=2  ·  10^−10^. As in other protocols Medina et al. [34]; Antonietti et al. [13]; Mauk and Ruiz [35]; and Medina and Mauk [36], the MF-DCN plasticity parameters have significantly lower values than the cortical plasticity, thus confirming the hypothesis that the effect of nuclear plasticities becomes meaningful on longer time scales.

The third plasticity (PC-DCN) gross exploration produced almost uniform results, if compared to the other two plasticities (Figure 4(c)). In the right area, the LTD is too high, and therefore, the PCs could not selectively inhibit the corresponding DCN. Even if more combination of LTP3 and LTD3 gave low cost function values, without defining a specific area, the parameter space near the global minimum was explored in the finer search. The finer exploration (Figure 4(c)) revealed homogeneous performances without particular spots of interest. The global minimum of this exploration corresponded to the parameters LTP3=10^−2^ and LTD3=10^−7^.

### 3.2. Simulated and Real Robot Performances

**Figure 5:**
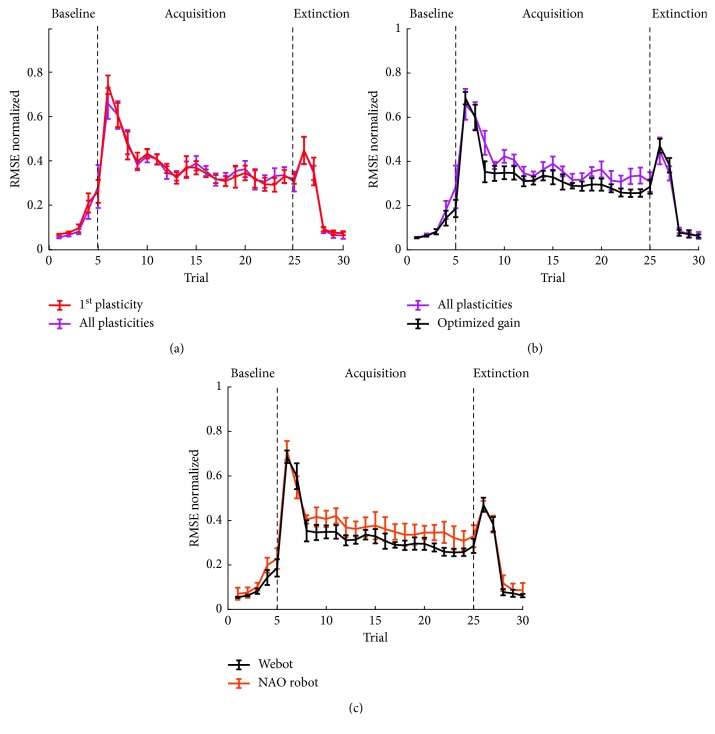
RMSE in different testing conditions. (a) Mean and SD of the RMSE computed for 10 tests with only the cortical plasticity optimized (in red) and after the optimization of the cortical and nuclear plasticities (in magenta). (b) Mean and SD of the RMSE computed for 10 tests after the optimization of the cortical and nuclear plasticities (in magenta) and after the optimization of the gain (in black). (c) Mean and SD of the RMSE computed for 10 tests after the optimization of the gain with Webot simulator (in black) and with NAO robot (in orange).

Having optimized the three learning rules, 10 tests were performed with only the cortical plasticity and with all the plasticities activated (Figure 5(a)). It becomes clear that the performance improvements given by the addition of the nuclear plasticities were negligible. As already mentioned, the main effect of nuclear plasticities could be seen on a longer time scale. It has been demonstrated [13] that the benefits of the nuclear plasticities can be verified in long paradigms, after more than 100 trials, possibly when more repeated sessions of acquisition and extinction are repeated. In addition, the possible improvements provided by the nuclear plasticities could be hidden by the nonnegligible variability between the 10 performed tests.

Once the three plasticities have been optimized, we proceeded with the tuning of the three joint gains. In the previous cases, all the joints used the same gain value of 0.012, theoretically derived from the foreseen maximum joint errors. The first rough exploration confirmed values near the ones already used. The results of the finer exploration identified the optimal gains as follows: Gain  1=0.005, Gain  1=0.013, and Gain  1=0.012. Since we have optimized the network plasticities with a fixed gain of 0.012, it is reasonable that the obtained gains are not very different from the original one.

Testing the SNN with optimized gains (Figure 5(b)), we obtained a generally lower error and a more stable trend in the acquisition phase, where the appropriate gain makes the intervention of the network more adequate in compensating the error, thus reducing the overcorrection effects leading to instability.

Having assessed the performance of the SNN in an almost ideal environment (the Webot simulator), we proceeded to test the model performance in the real world with NAO robot. The ten tests performed with NAO robot (Figure 5(b)) showed a proper correction in the acquisition phase, the after-effect at the beginning of the extinction phase, and a good extinction in the last trials. As expected, the Webot simulator performed slightly better than NAO robot, with smaller variance. However, the performance obtained with the NAO robot was still similar, with a good error reduction and similar physiological behaviour. Given the higher variability with the NAO robot, it would be even more difficult to notice differences between the performances of a SNN equipped with the cortical plasticity or with multiple plasticities.

### 3.3. Transfer Learning Performances

**Figure 6:**
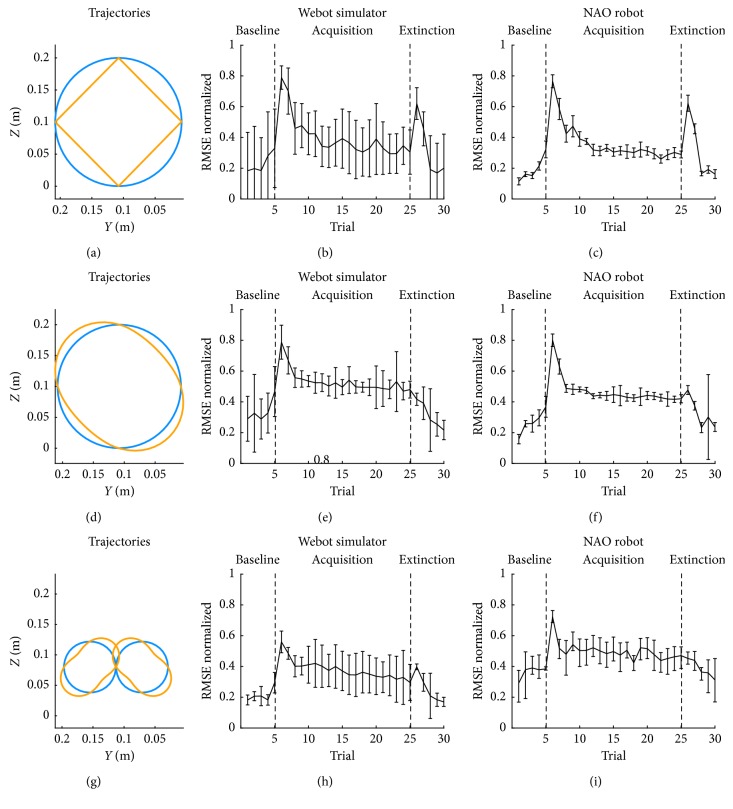
Transfer learning performances. (a, d, g) Ideal (blue) and perturbed (yellow) Cartesian trajectories in three cases: square, oval, and infinite, respectively. (b, e, h) Mean and SD of the RMSE computed for 10 tests with Webot simulator for the respective trajectories. (c, f, i) Mean and SD of the RMSE computed for 10 tests with NAO robot for the respective trajectories.

We wanted to test the transfer learning capabilities of the proposed SNN controller; we thus challenged the optimized SNN with three different ideal and perturbed trajectories (Figures 6(a), 6(d), and 6(g)). For each trajectory, we have adapted the gain values proportionally to the maximum error of every joint. Then, we tested the different shapes in Webot simulator (Figures 6(b), 6(e), and 6(h)) and in the NAO robot (Figures 6(c), 6(f), and 6(i)).

In the Webot simulator tests, comparing the error trends over time with the one obtained using the original trajectory, it is possible to notice a slightly higher variability exhibited by the oval trajectory, while the infinite and square trajectories performances were similar to the one obtained with the original perturbation. However, in all the cases, the overall performances were similar to the one achieved with the training trajectory.

In the NAO robot tests, differently from what was obtained with the Webot simulator, the worst performance was obtained with the infinite trajectory. This result is justified being the infinite trajectory, the one with the lower angular errors, with values more affected by the overall noise. Therefore, the SNN was less efficient and could not perform as well as in the other trajectories.

### 3.4. Network Enhancement

**Figure 7:**
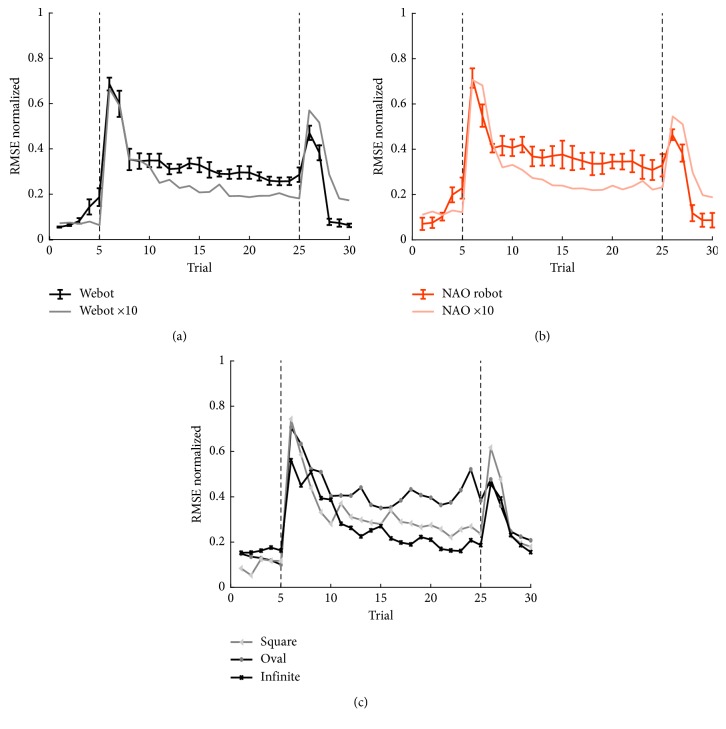
RMSE with the enhanced SNN. (a) Mean and SD of the RMSE computed for 10 tests with Webot simulator with the standard network (in black) and a single test with the enhanced tenfold SNN (in grey). (b) Mean and SD of the RMSE computed for 10 tests with NAO robot with the standard network (in orange) and a single test with the enhanced tenfold SNN (in light orange). (c) Mean and SD of the RMSE computed for three single tests performed with Webot simulator and with the three additional trajectories: square (light grey), oval (grey), and infinite (black).

One of the limits of the SNN used so far is the low number of output cells (DCN) which limits the resolution of the correction. As a result, the cerebellar correction on the joint angular values was jerky. To compensate for this effect, we tested a larger version (ten times larger) of the same network. All parameters have been kept the same as the ones used with the normal-sized network, and the gain has been reduced by a factor ten to match the increased number of output cells. A side effect of the increased size of the network is the loss of the real-time property, due to a larger number of spikes to be processed. Therefore the larger network was tested with both Webot simulator (Figure 7(a)) and NAO robot (Figure 7(a)) for a single test, instead of the usual 10 tests. The main improvements with respect to the normal-sized network were the initial error in the baseline phase, which remains around zero, and the lower and more stable RMSE in the acquisition phase. The other difference is in the extinction phase where the higher correction produces a higher overcompensation effect, and it requires more time to return to the initial state with respect to the normal-sized network.

The transfer learning capabilities were maintained in the larger SNN, also in these cases with smoother and more stable corrections of the joint errors in all the three additional trajectories. It is possible to notice that the enhancement of the SNN, augmenting the resolution of the network, made it slower in the adaptation processes. However, the parameter tuning carried out using the original SNN could be reused in the larger SNN, without having to rerun the optimization process (which would be unfeasible, given the extended computational loads of the larger SNN).

### 3.5. Neural Behaviour

We have also evaluated the network activity. The spikes generated by all the cells have been recorded during the testing phases and they could be analysed to verify how the errors affected the activity of the neuronal populations over the trials. The MFs kept an almost constant frequency over all the trials, with values comprised between 44 and 47 Hz. The GrCs were also almost constant with a frequency between 6 and 7 Hz; given the high number of these cells, their monitoring was quite challenging, and therefore their spike data were not collected in all the tests. PCs, IO, and DCN are the cells that explain the behaviour of the network, and from their variation in frequency, we can evaluate the physiological similarity of our system with a real biological one.

For every test, we recorded the ideal and real joint values, together with the actual Cartesian trajectory performed by the robot hand. Here, we report the network activity and the relative Cartesian and angular trajectories for the circle trajectory perturbed by an additional load application at the robot hand using the NAO robot and the enhanced SNN. Analyzing the salient trials of the protocol (see also the video provided as Supplementary Materials (available here)), it is possible to notice how the network activity shapes the robot behaviour and vice versa.

**Figure 8:**
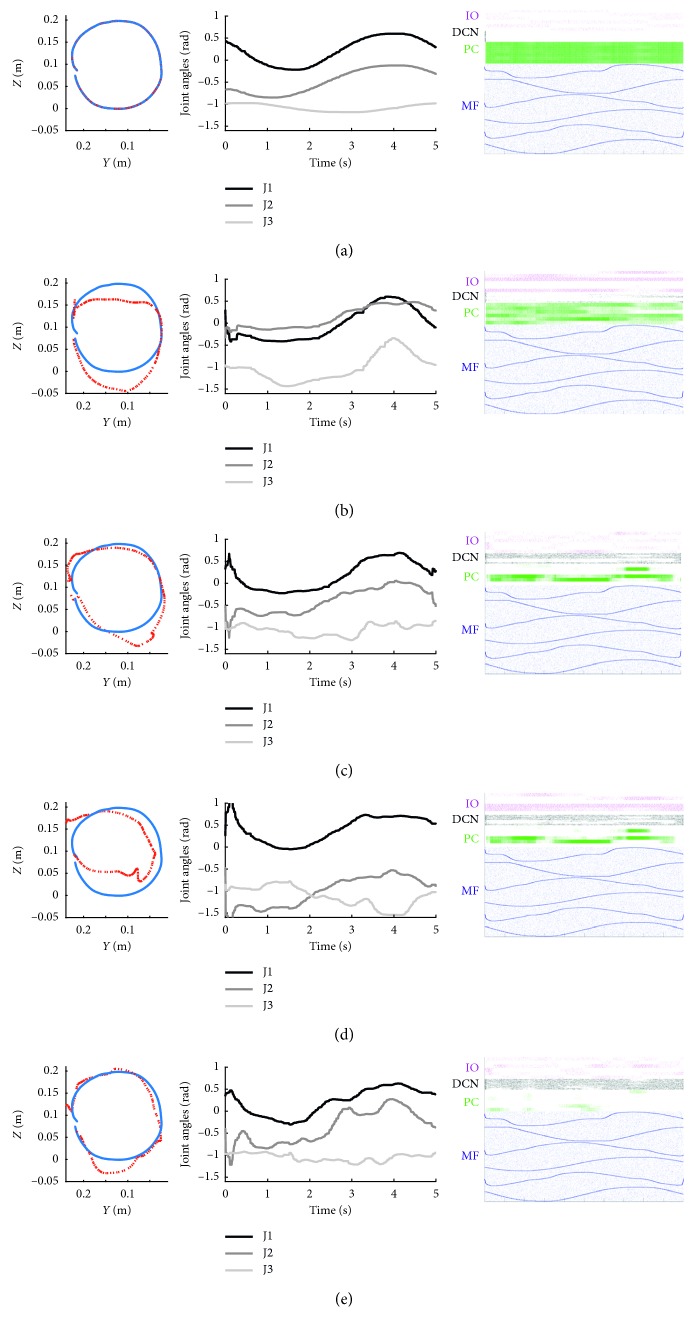
Cartesian and joint trajectories with the associated network activity for salient trials. Each row corresponds to a specific salient trial of the protocol: (a) Trial 1, when the test and the baseline phase starts; (b) Trial 6, when the acquisition phase starts; (c) Trial 25, the last trial of the acquisition phase; (d) Trial 26, the first trial of the extinction phase; (e) Trial 30, the last extinction trial and the last trial of the test. In each row, the first column represents the Cartesian trajectory in the *y-z* plane, where the blue line is the ideal trajectory (without perturbation, as in Trial 1) and the red line is the actual trajectory performed during that trial. The second column represents the three joint trajectories (joints 1–3 in black, grey, and light grey, respectively) performed during the trial. The third column represents the raster plots of the neural spikes produced by the SNN during that trial (MFs, PCs, DCNs, and IOs in blue, green, black, and magenta, respectively).

In the first trial of the baseline phase (Figure 8(a)), the robot is performing a correct trajectory, therefore IO activity is low, and the PCs are firing without restrictions. As a result, the output from the DCN is almost null.

In the first trial of the acquisition phase (Figure 8(b)), the robot hand is deviated by the additional load attached. The increased joint errors trigger the IO activity which rises consistently, although the IO population has a generally low frequency (<10 Hz). However, PC activity is still high, inhibiting the DCN. In the course of the trials the consistent activity of the IO reduces the PC's activity, reaching a point in which the DCN is free to fire as in the last trial of the acquisition (Figure 8(c)) where the PCs are selectively silent and the control signal generated by the DCN rises to compensate for the errors. From the Cartesian trajectories, the effect of the compensation in both the higher and the lower-left parts of the circumference is visible.

In the first trial of the extinction phase (Figure 8(d)), the additional load is removed, but the SNN network is still compensating for the error learned. This behaviour, generating errors in the opposite direction with respect to the acquisition phase errors, is proper of the cerebellar adaptation and it is called after-effect. In the last trial of the extinction phase (Figure 8(e)), we can observe that the after-effect has been canceled, and the performed trajectory is nearer to the desired one. However, observing the neural activity, it is possible to notice a nonphysiological behaviour. Normally, one would expect the change in sign from the IO to trigger the LTP effect on the PC thus inhibiting again the DCN cells. Here, however, there is a further inhibition of the PCs, this time of the opposite sign, which triggers the response of the DCNs of opposite sign, which were not firing until the beginning of the extinction phase. As a result, the absence of correction is caused by the cancellation of two opposite effects instead of a return to the initial DCN silence.

This unexpected result is probably due to the brevity of the protocol (only 20 acquisition trials) and to the cost function used for the parameter tuning, which rewarded higher values of LTD1 with respect to LTP1 for the cortical plasticity. This is visible from the steep slope of the RMSE in the first acquisition trials (Figure 3(c)). The low LTP1 values are not enough to restore the initial state of the network with only 5 trials for the extinction phase, and therefore the optimization process rewarded a configuration where LTD1 compensated for the error in opposite sign with the activity of the DCN of opposite sign (antagonist activity) instead of decreasing the current (agonist) DCN activity. This effect led to the nullification of the agonist and antagonist neuron activity, with a net output near zero (i.e., near the desired network output during the extinction phase). This result suggests that even with an imbalance in the cortical LTP/LTD ratio, a system can be still able to learn and extinguish a motor adaptation. This hypothesis should be tested by ad hoc experiments where cortical LTP mechanisms have to be blocked or impaired (similar to what was done with mutant mice by Schonewille et al. [37]).

## 4. Conclusions

In this work, we aimed at the integration of a bioinspired SNN in a NAO robot controller. In particular, the cerebellum has been chosen as the neural structure to emulate for its critical role in motor learning task. The integration of a cerebellar structure in a robot could help in developing new paradigms and ways to perform robotic control in different motor tasks.

Our work was based on previous ones; here, we introduced a larger version of the network, able to control simultaneously three DoFs instead of a single one. This allowed the robot to be tested in a more complex task, using the SNN in an adaptation of the upper limb perturbed reaching protocol, usually used to test the cerebellar learning properties.

We obtained positive results, and the SNN performed well when tested with different trajectories, showing the cerebellar property of transfer learning (i.e., generalizability). The possibility to adapt to different motor task is a fundamental property for the aim of bioinspired robot controllers which will have to deal with different kinds of motor tasks.

One of the main limitations of our network was the low resolution in the output control signal. Tests carried out with a larger network (ten-fold). With this network, the larger number of DCN could produce a smoother output and deal better with small errors.

Further investigation on this network can be performed with the other typical cerebellar paradigms. As in [12], this SNN maintains a general purpose for other cerebellar related tasks. Therefore it would be possible to adapt both the network and the robot to Pavlovian conditioning or vestibulo-ocular reflex protocols.

The increase in the number of controlled joints as well as the good performances obtained with our test, suggests that a larger network would be ideal to tackle this kind of motor tasks. This is especially true if the SNN has to control a real system, in a world rich of unpredictable errors that lowered network performances. The real-time simulation of such large-scale SNN, to develop a real bioinspired controller for a physical robot, could be obtained uniquely by means of highly parallel computing (e.g., GPU) or neuromorphic hardware. This could help in the developing of better strategies of robot control, capable of motor learning and event correlation that would meet practical use in many fields, from the industry to artificial intelligence applications.

## Data Availability

The data generated by the optimization process and by simulations done with the Webot simulator and with the NAO robot have been deposited in the Harvard Dataverse repository. In addition, MATLAB scripts which reproduce all the figures presented in this work are provided (DOI: https://doi.org/10.7910/DVN/HEPECM).

## Supplementary Materials

Supplementary Materials A supplementary video is provided, which illustrates the 3D motion task performed by the NAO robot, driven by the SNN controller, in the three experimental phases: 5 baseline trials (in an unperturbed condition); 20 acquisition trials (with a perturbation caused by a virtual load attached at NAO hand); and 5 extinction trials (where the additional load is removed).
