# Corrigendum to “Control of a Humanoid NAO Robot by an Adaptive Bioinspired Cerebellar Module in 3D Motion Tasks”

**DOI:** 10.1155/2020/2390349

**Published:** 2020-10-22

**Authors:** Alberto Antonietti, Dario Martina, Claudia Casellato, Egidio D'Angelo, Alessandra Pedrocchi

**Affiliations:** ^1^Department of Electronics, Information and Bioengineering, Politecnico di Milano, Milano, Italy; ^2^Department of Brain and Behavioral Sciences, University of Pavia, Pavia, Italy; ^3^IRCCS Mondino Foundation, Pavia, Italy

In the article titled “Control of a Humanoid NAO Robot by an Adaptive Bioinspired Cerebellar Module in 3D Motion Tasks” [1], there was an error in Dr. Egidio D'Angelo's affiliation. The corrected authors list and affiliations are shown above.
